# The relationship between preference-based health-related quality of life and lifestyle behavior: a cross-sectional study on a community sample of adults who had undergone a health check-up

**DOI:** 10.1186/s12955-020-01518-6

**Published:** 2020-08-03

**Authors:** Shinichi Noto, Osamu Takahashi, Takeshi Kimura, Kensuke Moriwaki, Katsunori Masuda

**Affiliations:** 1 Department of Occupational Therapy, Niigata University of Health and Welfare, Niigata, Japan; 2 Graduate School of Public Health, St. Luke’s International University, Tokyo, Japan; 3 Center for Preventive Medicine, St. Luke’s International Hospital, Tokyo, Japan; 4 Comprehensive Unit for Health Economic Evidence Review and Decision Support (CHEERS), Research Organization of Science and Technology, Ristumeikan University, Kyoto, Japan

## Abstract

**Background:**

Preference-based Health-Related Quality of Life (HRQL) is one of the most important indicators for calculating QALY (Quality-Adjusted Life Years) in a cost-effectiveness analysis. This study aimed to collect data on healthy individuals’ HRQL based on the preferences of Japanese people who had undergone a comprehensive health check-up, and to examine the influence of relevant factors, such as blood biochemical data and lifestyle behavior.

**Methods:**

We conducted a cross-sectional study targeting people who had undergone a comprehensive health check-up in 2015. Participants were asked to respond to a medical interview sheet. We then examined the utility value, as well as lifestyle habits such as alcohol intake, smoking, and exercise. HRQL was examined using EQ-5D-5L. Using a multiple regression analysis, we examined the influence of related factors, such as lifestyle and biochemical test data.

**Results:**

We collected 2037 responses (mean age = 54.98 years; 55.0% female). The average preference-based health-related HRQL was 0.936 ± 0.087. A total of 1167 people (57.2%) responded that they were completely healthy. The biochemical test data that were recognized to correlate with HRQL were hemoglobin, total cholesterol, creatinine, all of which were weak (*r* = − 0.045–0.113). The results of multiple regression analysis showed that significant facts were: being female, age (≧70 year-old), drinking alcohol (sometimes), activity (very often), and lack of sleep.

**Conclusions:**

The HRQL of participants who had undergone a comprehensive health check-up was generally high, and only declined for those over 70 years of age. It is suggested that preference-based HRQL is related to physical activity, and that decrease of activity and lack of sleep leads to a decrease in HRQL.

## Introduction

Preference-based Health-Related Quality of Life (HRQL) is one of the most important indicators, along with cost, for calculating QALY (Quality-Adjusted Life Years) in a cost-effectiveness analysis. Due to its application in the Health Technology Assessment (HTA) policy, its importance has increased. Since the HTA began in Japan in 2017, guidelines have been developed [1], according to which the evaluation tool recommended for Japan requires a scoring algorithm based on data investigated domestically. Currently, only EQ-5D satisfies this guideline.

EQ-5D was developed by the EuroQol Group, and two scoring algorithms, EQ-5D-3L [2] and EQ-5D-5L [3], have been developed for Japan. Since EQ-5D-5L is said to be less susceptible to the ceiling effect recognized in EQ-5D-3L [4–6], EQ-5D-5L is expected to be utilized. However, there is little norm data on healthy subjects. In a survey of healthy subjects, it has been reported that factors such as, being over 70 -years -old, having low-income, and a short education period, decrease HRQL [7]. However, other factors, such as age, income, and education, have not been shown to affect HRQL. If the relationship between lifestyle factors and HRQL is clarified, it can be used as basic data to judge the economic effects of public health policy in Japan, such as prevention of long-term care and medical examinations in the future. In addition, since this study is being conducted in Tokyo, the capital city, in the future it will be possible to compare assessment study populations with community populations.

In Japan, it is common practice for healthy people to check their health status annually by participating in a comprehensive health check-up [8]. This check-up includes a blood biochemical test as well as a chest X-ray, confirmation of lifestyle behavior, medical interview, etc. In this study, our aim was to examine HRQL by applying EQ-5D-5L to people who had undergone the above-mentioned comprehensive health check-up, and to investigate its relationship to lifestyle and blood biochemical data.

## Methods

### Sampling design

We targeted people who had undergone a comprehensive health check-up from January to March 2015, at the Clinic and Center for Preventive Medicine affiliated with St. Luke’s International Hospital in Tokyo. The hospital was located in the center of the capital, so the population surveyed was mostly urban residents. A total of 3800 participants had undergone the health check-up. Prior to the examination, medical interview sheets were sent to the subjects by mail. The sheets included a lifestyle survey and an EQ-5D-5L. These sheets were then collected on the day of the examination. Subjects had to be over 21 years of age and able to answer the medical interview sheet on their own.

### EQ-5D-5L

The EQ-5D-5L is a generic preference-based measure of HRQL developed by the EuroQol Group [7]. The five dimensions are as follows: mobility (MO), self-care (SC), usual activities (UA), pain/discomfort (PD), and anxiety/depression (AD). Each has five levels [9]. The Japanese version of the EQ-5D-5L was used in this study; therefore, the responses obtained were converted to HRQL scores based on Japanese value sets [3].

### Medical interview sheet

Through self-report, the medical interview sheet confirmed whether or not the person was currently undergoing treatment for a disease (Hypertension, Hyperlipidemia, Diabetes, Gout, Glaucoma, Cataract, Depression, Reflux Esophagitis, Osteoporosis, Fibroid, and/or Breast Cancer). Furthermore, people were asked about the following lifestyle habits: alcohol intake, 3 levels, (level 1, “Never (not drink at all),” level 2, “Sometimes (2-3 times a week),” level 3, “Habitually”); smoking, 3 levels (level 1, “Never (have never smoked at all),” level 2, “Active (smoke on a daily basis),” level 3, “Former smoker”); lack of exercise, 2 levels (level 1, “No,” level 2, “Yes”); physical activity, 4 levels (level 1, “Very often,” level 2, “Usually,” level 3, “Not so active,” level 4, “Too little”); exercise habit, 4 levels (level 1, “Almost daily,” level 2, “3-5times/week,” level 3, “1-2times/week,” level 4, “Too little”); and sleep, 2 levels (level 1, “Well,” level 2, “Lack of sleep”). Physical activity comprises subjective activity in daily life, while exercise habit comprises sweaty exercise for more than 20 min.

### Health check-up

In the comprehensive health checkup, body mass index (BMI), body fat, blood pressure was measured, and biochemical examination of blood was performed. The blood biochemical test examined the hemoglobin, blood glucose, HbA1c, low-density lipoprotein (LDL), high-density lipoprotein (HDL), total cholesterol, neutral fat, thyroid-stimulating hormone (TSH), free thyroxine 4 (FT4), glutamate oxaloacetate transaminase (GOT), glutamate pyruvate transaminase (GPT), creatinine, and estimated glomerular filtration rate (EGFR). These blood biochemical examination findings were related to lifestyle-related diseases such as hypertension and heart disease. These series of examinations comprised the basic elements of the complete health checkup and were performed by the clinician, with the cost borne by the study subjects.

### Statistical analysis

A summary of HRQL scores was calculated by gender, age group (21–39, 40–49, 50–59, 60–69, and ≧70 year-old) and lifestyle factors, and scores were then compared using variance analysis. To detect the influence of demographic factors and lifestyle factors on the HRQL scores, these variables were added to an analysis of variance (ANOVA). The influence of demographic characteristics and lifestyle factors on HRQL was determined using multiple regression analyses. Sex, age, drinking, smoking, physical activity and sleep, with the relevant dummy variables, were included as covariates in a multiple regression model with total HRQL score as the outcome. Exercise habit was not included in this model due to potential collinearity with physical activity. The significance level was set at 0.05. Statistical analyses were performed using STATA 14.0. This study was approved by the Ethics Committee of Niigata University of Health and Welfare (No. 17560–150,205) and the Institutional Review Board of St. Luke’s International Hospital (No.15-R086). Informed consent was obtained from all individual participants included in the study.

## Results

### Demographic and health characteristics

Table 1 shows the demographic and health characteristics of the subjects. Of the 3800 people who mailed medical interview sheets, 2104 (response rate: 55.0%) were collected, of which 67 were found to be incomplete, and 2037 were included in the analysis. Of the total, 55% were female subjects (mean age = 54.98 years). The average BMI was 22.5 ± 3.3 kg/m^2^ and the average Body Fat was 24.3 ± 6.5%. Blood pressure and blood biochemical data were all within the standard value as average. Regarding self-reported chronic diseases, 300 people (14.7%) reported having high blood pressure, 276 (13.5%) reported having hyperlipidemia, 105 (5.1%) reported diabetes, 98 (4.8%) reported having uterine myoma, and 86 (4.2%) had gout.

**Table 1:** Demographic and health characteristics of the study participants

Characteristic	*n* (%) or Mean ± SD^a^	95%CI
Sex		
Male	916 (45.0)	
Female	1121 (55.0)	
Age^a^	55.0(11.7)	
21–39	163 (8.0)	
40–49	546(26.8)	
50–59	650(31.9)	
60–69	433(21.3)	
70<	245(12.0)	
BMI^a^	22.5 ± 3.3	22.5–22.6
Body Fat (%)^a^	24.3 ± 6.5	24.0–24.6
Blood Pressure^a^		
SBP	120.3 ± 16.3	119.6–121.0
DBP	73.5 ± 11.6	73.0–74.0
Hemoglobin	14.0 ± 1.3	13.9–14.0
Blood glucose	101.2 ± 14.1	100.6–101.9
HbA1c	5.7 ± 0.5	5.7–5.7
LDL	123.1 ± 29.5	122.1–124.7
HDL	65.9 ± 16.8	65.1–66.6
Total cholesterol	209.9 ± 33.1	208.6–211.6
Neutral fat	92.7 ± 66.4	89.7–95.5
TSH	2.5 ± 1.8	2.4–2.6
FT4	1.2 ± 0.0	1.2–1.3
GOT	21.9 ± 8.4	21.5–22.2
GPT	21.7 ± 14.0	21.0–22.3
Creatinine	0.7 ± 0.2	0.71–0.72
EGFR	78.8 ± 14.2	78.2–79.4
Self-reported chronic diseases		
Hypertension	300(14.7)	
Hyperlipidemia	276(13.5)	
Diabetes	105 (5.1)	
Gout	86 (4.2)	
Glaucoma	83 (4.1)	
Cataract	64 (3.1)	
Depression	23 (1.1)	
Reflux esophagitis	63 (3.1)	
Osteoporosis	70 (3.4)	
Fibroid	98 (4.8)	
Breast cancer	60 (2.9)	

^a^*SD* Standard deviation, *CI* Confidence interval, *BMI* Body mass index, *SBP* Systolic blood pressure, *DBP* Diastolic blood pressure, *LDL* Low-density lipoprotein, *HDL* High-density lipoprotein, *TSH* Thyroid-stimulating hormone, *FT4* Free thyroxine 4, *GOT* Glutamate oxaloacetate transaminase, *GPT* Glutamate pyruvate transaminase, *EGFR* Estimated glomerular filtration rate

### HRQL score and relation to demographic and lifestyle factors

Table 2 shows the EQ-5D-5L score for each demographic characteristic and health habit. The average EQ-5D-5L index score of male subjects was 0.946 ± 0.081, which was higher than that of female subjects at 0.927 ± 0.090 (*p* < 0.001). There was no statistically significant difference regarding age group (*p* = 0.067).

**Table 2:** EQ-5D-5L index score by characteristics and lifestyle factors of the study participants

	n(%)	*EQ-5D-5L* *Index score* (Mean ± SD)	*P* value	Full health reporting n(%)	*EQ-5D-5L* *VAS* (Mean ± SD)	*P* value
All		0.936 ± 0.087		1167(57.2)	79.9 ± 12.3	
Sex						
Male	916 (45.0)	0.946 ± 0.081	< 0.001	586(64.2)	79.9 ± 12.4	0.774
Female	1121 (55.0)	0.927 ± 0.090		581(51.8)	80.0 ± 12.2	
Age						
21–39	163 (8.0)	0.934 ± 0.094	0.067	97(59.5)	75.7 ± 16.2	< 0.001
40–49	546(26.8)	0.940 ± 0.083		334(61.2)	79.4 ± 12.6	
50–59	650(31.9)	0.937 ± 0.080		363(55.8)	79.9 ± 11.3	
60–69	433(21.3)	0.936 ± 0.082		241(55.7)	81.0 ± 11.6	
70<	245(12.0)	0.921 ± 0.112		132(53.9)	82.4 ± 11.4	
Drinking						
Never	796(39.1)	0.923 ± 0.098	< 0.001	410(51.5)	79.9 ± 12.3	0.995
Sometimes	299(14.7)	0.943 ± 0.076		181(60.5)	79.9 ± 12.8	
Habitually	933(45.8)	0.944 ± 0.079		572(61.3)	79.9 ± 12.2	
Smoking						
Never	1293(63.5)	0.934 ± 0.087	0.162	727(56.2)	79.9 ± 12.0	0.505
Active	161(7.9)	0.948 ± 0.079		104(64.6)	81.0 ± 11.2	
Former-smokers	572(28.1)	0.936 ± 0.089		332(58.0)	79.7 ± 13.2	
Lack of Exercise						
No	559(27.4)	0.956 ± 0.069	< 0.001	380(68.0)	83.3 ± 11.4	< 0.001
Yes	1451(71.2)	0.928 ± 0.092		774(53.3)	78.6 ± 12.4	
Physical Activity						
Very often	188(9.2)	0.967 ± 0.062	< 0.001	141(75.0)	84.9 ± 12.9	< 0.001
Usually	1055(51.8)	0.944 ± 0.076		636(60.3)	81.3 ± 11.3	
Not so active	599(29.4)	0.918 ± 0.098		293(48.9)	77.2 ± 12.2	
Too little	184(9.0)	0.912 ± 0.111		93(50.5)	75.7 ± 14.3	
Exercise habit						
Almost daily	258(12.7)	0.944 ± 0.088	0.0011	164(63.6)	83.3 ± 12.5	< 0.001
3–5/week	404(19.8)	0.945 ± 0.079		253(62.6)	81.1 ± 12.1	
1–2/week	787(38.6)	0.936 ± 0.083		436(55.4)	79.6 ± 11.3	
Too little	577(28.3)	0.925 ± 0.096		310(53.7)	78.1 ± 13.3	
Sleeping						
Well	1228(60.3)	0.952 ± 0.077	< 0.001	817(66.5)	82.2 ± 11.7	< 0.001
Lack of sleep	738(36.2)	0.909 ± 0.096		316(42.8)	76.3 ± 12.5	

We further examined the relationship between lifestyle and HRQL score. Regarding alcohol intake, the mean score for “never” was 0.923 ± 0.098, while higher values were shown (*p* < 0.001) for who drank alcohol “sometimes” (0.943 ± 0.076) and “habitually” (0.944 ± 0.079) (*p* < 0.001). Regarding lack of exercise, the highest mean scores were for “no” at 0.956 ± 0.069, with “yes” at 0.928 ± 0.092 (*p* < 0.001). A difference was also observed for daily activities, with a mean score of 0.967 ± 0.062 for “very often” in contrast to 0.912 ± 0.111 for “too little.” Finally, differences were found in exercise habit (*p* = 0.0011) and lack of sleep (*p* < 0.001).

Data of the visual analogue scale (VAS) were also analyzed. There were no differences in gender (*p* = 0.774), but differences were observed among age groups (*p* < 0.001), lack of exercise (*p* < 0.001), exercise habit (*p* < 0.001), and sleep patterns (*p* < 0.001).

### Correlation with HRQL scores and health check-up data

Table 3 shows the correlation between EQ-5D-5L index scores and blood biochemical data. A statistically significant correlation was observed for body fat (*r* = − 0.077), hemoglobin (*r* = 0.113), total cholesterol (*r* = − 0.045) and creatinine (*r* = 0.089), but the correlation was weak for all of them. BMI, body fat, HDL, GPT, and EGFR were also observed to have a correlation to VAS.

**Table 3:** Correlation between EQ-5D-5L index score and medical checkup data

Characteristic	R^*^	
	*EQ-5D-5L Index*	*EQ-5D-5L VAS*
BMI	−0.015	− 0.050^*^
Body Fat (%)	− 0.077^**^	− 0.067^**^
Blood Pressure		
SBP	− 0.033	− 0.009
DBP	0.016	−0.028
Hemoglobin	0.113^**^	− 0.001
Blood glucose	− 0.037	0.008
HbA1c	−0.035	0.037
LDL	−0.036	−0.028
HDL	−0.022	0.061^**^
Total cholesterol	− 0.045^*^	0.005
Neutral fat	0.007	−0.030
TSH	−0.030	0.020
FT4	0.004	−0.018
GOT	−0.009	−0.020
GPT	−0.007	− 0.066^**^
Creatinine	0.089^**^	0.015
EGFR	− 0.024	− 0.076^**^

^**^p < 0.01, ^*^p < 0.05

*BMI* Body mass index, *SBP* Systolic blood pressure, *DBP* Diastolic blood pressure, *LDL* Low-density lipoprotein, *HDL* High-density lipoprotein, *TSH* Thyroid-stimulating hormone, *FT4* Free thyroxine 4, *GOT* Glutamate oxaloacetate transaminase, *GPT* Glutamate pyruvate transaminase, *EGFR* Estimated glomerular filtration rate

### HRQL score and relation to socio-demographic and lifestyle factors

Finally, Table 4 shows the relationship between EQ-5D-5L scores, demographic characteristics, and lifestyle habits as determined by the multiple regression analysis. Female subjects had lower HRQL scores than males (coefficient − 0.019, *p* < 0.001). Moreover, subjects over 70 years of age had lower scores than those under 70 years of age (coefficient − 0.036, *p* < 0.001). Regarding alcohol intake, both “sometimes” (coefficient 0.014, *p* < 0.001) and “habitually” (coefficient 0.012, *p* < 0.001) scored higher than “never.” Additionally, daily activities were indicated as factors causing a decrease, in contrast with “very often,” “usually” (coefficient − 0.018, *p* = 0.011), “not so active” (coefficient − 0.042, *p* < 0.001), and “too little” (coefficient − 0.046, *p* < 0.001). Lack of sleep was also indicated as a factor that lowers HRQL scores (coefficient − 0.039, *p* < 0.001).

**Table 4:** Relationship between EQ-5D-5L index score and demographic characteristics and health customs

	*N*(%)	Coefficient	*P* value	95%CI
Intercept		0.991	< 0.001	0.970–1.012
Sex				
Male	916 (45.0)	–	–	–
Female	1121 (55.0)	**−0.019**	< 0.001	− 0.028--0.010
Age				
21–39	163 (8.0)	–	–	–
40–49	546(26.8)	0.003	0.663	−0.012-0.017
50–59	650(31.9)	−0.003	0.728	−0.017-0.012
60–69	433(21.3)	−0.013	0.125	−0.029-0.002
≧70	245(12.0)	**−0.035**	< 0.001	−0.052--0.017
Drinking				
Never	796(39.1)	–	–	–
Sometimes	299(14.7)	**0.014**	0.017	0.003–0.025
Habitually	933(45.8)	**0.013**	0.010	0.010–0.016
Smoking				
Never	1293(63.5)	–	–	–
Active	161(7.9)	−0.002	0.807	−0.016-0.012
Former smoker	572(28.1)	−0.009	0.249	−0.023-0.006
Physical Activity				
Very often	188(9.2)	–	–	–
Usually	1055(51.8)	**−0.022**	0.001	−0.035--0.009
Not so active	599(29.4)	**−0.049**	< 0.001	−0.062--0.035
Too little	184(9.0)	**−0.053**	< 0.001	−0.070--0.036
Sleeping				
Well	1228(60.3)	–	–	–
Lack of sleep	738(36.2)	**−0.039**	< 0.001	−0.047--0.031

Adjusted R2: 0.119. “-” shows reference group. Boldness suggests statistical significance (i.e., *P* < 0.05)

## Discussion

The results of this study demonstrated that the preference-based HRQL of healthy subjects who had undergone health checkups is related to demographic characteristics and lifestyle factors and is influenced by blood biochemical data. First, it was clear that the factor of aging did not lower the HRQL of people who live independently and receive health examinations. A medical check-up is a comprehensive test that uses blood biochemical tests to check for the onset of disease, so those who receive it are basically healthy. However, HRQL declines for the elderly over 70 years old. This is similar to the findings of Shiroiwa et al. [7]. Regarding differences due to gender, women showed lower values than their male counterparts. This is also similar to finding of Shiroiwa et al. [7]. This trend has been confirmed in South Korea [10], Germany [11], Poland [12], and Spain [13], but gender differences have not been observed in the US [14], Singapore [15], or southern Australia [16]. However, there are few research results showing low values for men, which may reflect men’s tendency to respond optimistically, at least to the HRQL using EQ-5D.

There was no strong influence on the relationship between the health data collected by the comprehensive health check-up and HRQL indicated by EQ-5D-5L. It can be considered that blood biochemical data do not directly affect the five dimensions of mobility, self-care, usual activities, pain/discomfort, and anxiety/depression, as indicated by EQ-5D.

The relationship between lifestyle and HRQL does have an impact, particularly alcohol intake, daily physical activity, exercise habit, and sleep patterns. Regarding alcohol intake, since people with drinking habits showed higher HRQL scores, this indicates that drinking does not lower HRQL. Drinking can be considered to have a positive influence on HRQL, since as indicated in several previous studies [17–19], moderate alcohol consumption has an effect of preventing cardiac disease. In South Korea, abstinence from alcohol consumption resulted in a higher quality of life for elderly people living in the community [20], while people in NYHA class I who consume moderate amounts of alcohol tend to have a better quality of life [21]. Since the present study targeted adults over 21 years old, moderate alcohol intake is considered to have a positive influence on HRQL for all generations. Paradoxically, however, there may have been some factors that lower HRQL more for non-drinkers than for those who do drink. The relationship between alcohol consumption and HRQL requires further investigation because there is insufficient evidence. In addition, an examination of daily physical activity shows that HRQL is lower for those who do not move their bodies often compared with those who do. Past studies and reviews have reported that physical activity produces a high quality of life [22–26]. However, many of these were profile-type scales, such as SF-36 and WHOQOL. Therefore, it is meaningful that this has also been confirmed for preference-based HRQL. Finally, lack of proper sleep was shown to lower HRQL. The relationship between sleep and HRQL has shown that moderate sleep of approximately 7 h brings a high quality of life, but HRQL declines with either too much or too little sleeping [27, 28]. In this study, we only investigated lack of sleep, but the results are similar to previous studies. As described above, various lifestyle factors are related to the HRQL score indicated by EQ-5D- 5 L, but their causal relationships could not be clarified. In the future, it is considered that it is necessary to verify of lifestyle factors on future HRQL, while clarifying the causal relation between lifestyle factors and HRQL in more detail.

One of the limitations of this study is that the sample size is small compared to other studies targeting healthy subjects. Moreover, the study was only conducted in one hospital. Further research should clarify the relationship between chronic disease and HRQL scores, by increasing the sample size and conducting a longer-term survey. We would also like to reduce any possible bias that may accrue from including so few participating facilities by conducting a multi-facilities collaborative research project.

## Conclusions

In this study, we showed the HRQL of healthy subjects as indicated by EQ-5D-5L through a comprehensive health check-up, and clarified the relationship between demographic characteristics, blood biochemical data, and lifestyle behavior. It was shown that the factors leading to a decline of HRQL are being female and over 70 years of age, while factors that keep HRQL high are alcohol intake, high levels of physical activity in daily life, and adequate sleep. The results of this study will provide good suggestions for public health policies, including care prevention.

## Supplementary information

**Additional file 1.**

## Data Availability

The datasets generated and analyzed during the current study are not publicly available due to reasons of data protection but are available from the Institute of Occupational Therapy, Niigata University of Health and Welfare, on reasonable request.
