# Supplementary material for: The relationship between preference-based health-related quality of life and lifestyle behavior: a cross-sectional study on a community sample of adults who had undergone a health check-up
Source: Health Qual Life Outcomes. 2020 Aug 3;18:267. doi: 10.1186/s12955-020-01518-6 (PMC7398297; doi:10.1186/s12955-020-01518-6)
Supplement: Supplementary file 1 — Additional file 1. [file 12955_2020_1518_MOESM1_ESM.pdf]

生活習慣問診票

１．飲酒について

〇現在の飲酒について

□習慣的に飲む　□ときどき飲む　□飲まない

２．たばこについて

〇現在の喫煙について

□吸っている　□吸わない　□過去に吸っていた

３．運動について

〇運動不足と思いますか

□思う　□思わない

〇日常における身体活動はどのくらいですか

□よく身体を動かしている　□普通に動いている　□あまり活動的でない　□ほとんど体を動かさない

〇運動習慣は（軽く汗ばむ運動を 20 分以上）

□ほとんど毎日　□週 3～5 日　□週 1～2 日　□ほとんどしない

４．睡眠について

□よく眠れる　□寝不足を感じる
